# Supplementary material for: Standardising Breast Radiotherapy Structure Naming Conventions: A Machine Learning Approach
Source: Cancers (Basel). 2023 Jan 17;15(3):564. doi: 10.3390/cancers15030564 (PMC9913464; doi:10.3390/cancers15030564)
Supplement: Supplementary file 1 [file cancers-15-00564-s001.zip › cancers-2029934-supplementary.pdf]

S1. Standarised Breast Radiotherapy Structures Names

Table S1. Classes utilized in each dataset.

Structure	Dataset 1	Dataset 2	Dataset 3	Dataset 4	Dataset 5
axilla ctv	-	-	X	X	X
axilla ptv	-	-	X	X	X
boost structure	-	-	-	X	X
breast ctv	-	X	X	X	X
breast ptv	-	X	X	X	X
chestwall ctv	-	X	X	X	X
chestwall ptv	-	X	X	X	X
combined lungs	X	X	X	X	X
combined structure	-	-	-	X	X
contralateral	X	X	X	X	X
control structure	-	-	-	X	X
exclude	-	-	-	-	X
heart	X	X	X	X	X
imc ctv	-	-	X	X	X
imc ptv	-	-	X	X	X
left lung	X	X	X	X	X
right lung	X	X	X	X	X
scf ctv	-	-	X	X	X
scf ptv	-	-	X	X	X
tumourbed ctv	-	X	X	X	X
tumourbed ptv	-	X	X	X	X
Total	5	11	17	20	21

S2. Experiments and Results

Table S2. (a) Experiments conducted in each case study.

Case study	Features	Type of the network
1	Text	Feed forward neural network
2	Dose	Feed forward neural network
3	Geometry	Feed forward neural network
4	Images	Convolutional neural network
5	Text + dose	Feed forward neural network
6	Text + geometry	Feed forward neural network
7	Text + images	Multi-input neural network
8	Geometry + dose	Feed forward neural network
9	Dose + image	Multi-input neural network
10	Geometry + image	Multi-input neural network
11	Text + geometry + dose	Feed forward neural network
12	Text + dose + image	Multi-input neural network
13	Text + geometry + image	Multi-input neural network
14	Geometry + dose +image	Multi-input neural network
15	Text + dose + geometry +image	Multi-input neural network

Table S2. (b) list of misclassified volumes predicted by the best performing model over the test dataset (study 5).

Structure name	Structure category/class	Predicted category/class name	Probability
PTV_RTAXILLA	axilla_ptv_approved	combined_structure	0.611028
SCLAV_PTV	scf_ptv_approved	exclude	0.704626
SCLAV_PTV	scf_ptv_approved	exclude	0.520875
RO_SEROMA	tumourbed_ctv_approved	exclude	1
PTV42,4old	exclude	breast_ptv_approved	0.975655
ChestwallAx_CTV	combined_structure	axilla_ctv_approved	0.81999
lung0.5	exclude	left_lung_approved	0.999727
PTV_NODES_02	breast_ptv_approved	exclude	0.717039
L_BTREAST_PTV	breast_ptv_approved	exclude	0.987708
LEVEL_3_NODE_CTV_BOOST	boost_structure	exclude	0.934735

Table S2. (c) list of misclassified samples by the best performing model over the extended test dataset.

Structure name	Structure category/class	Predicted category/class name	Probability
PTV_RTAXILLA	axilla_ptv_approved	combined_structure	0.669003
SCLAV_PTV	scf_ptv_approved	breast_ptv_approved	0.889554
PTV42,4	breast_ptv_approved	exclude	0.536556
PTV_EVAL_STARS	breast_ptv	exclude	0.984457
SCLAV_PTV	scf_ptv_approved	breast_ptv_approved	0.692799
RO_SEROMA	tumourbed_ctv_approved	exclude	1
PTV42,4old	exclude	breast_ptv_approved	0.981575
PTV_TUMOURBEDBOOST	boost_structure	tumourbed_ctv_approved	0.729915
EXT-PTV	breast_ptv	exclude	0.999907
ChestwallAx_CTV	combined_structure	breast_ctv_approved	0.554923
ChestwallAx_PTV	combined_structure	breast_ptv_approved	0.557953

lung0.5	exclude	left_lung_approved	0.992461
CTV_IP_NODES	breast_ctv	exclude	0.999103
PTV60_RING	exclude	breast_ptv_approved	0.786016
L_BTREAST_PTV	breast_ptv_approved	exclude	0.952107
PTV42,4_EVAL	breast_ptv	exclude	0.510468
PTVLD_EVAL	breast_ptv	exclude	0.945179
PTVLD_EVAL	breast_ptv	exclude	0.892079
REG_CTV_L_SCV	scf_ctv	exclude	0.999954

Table S2. (d) Time taken to train each model in each dataset (in seconds).

**case study**	**dataset 1**	**dataset 2**	**dataset 3**	**dataset 4**	**dataset 5**
text	120	196	129	303	465
dose	125	191	204	293	479
geometry	124	183	242	301	478
images	933	2346	2481	3872	9782
text_dose	62	106	193	272	432
text_geometry	88	155	274	262	465
text_image	685	1130	1603	2551	3931
geometry_dose	117	192	276	289	472
dose_image	1141	910	740	2310	8332
geometry_image	2529	1831	1660	4694	8064
text_geometry_dose	82	168	260	240	382
text_dose_image	811	1336	1665	1286	3034
text_geometry_image	830	992	1274	1361	3914
geometry_dose_image	503	1510	2215	4148	4673
text_dose_geometry_image	366	1000	1067	1529	3895

Table S2. (e) Classification report of the best performing model over the extended dataset (text+dose+image)

**classes**	**f1-score**	**precision**	**recall**	**support**
axilla_ctv_approved	1	1	1	27
axilla_ptv_approved	0	0	0	1
boost_structure	0.91	1	0.83	6
breast_ctv_approved	1	1	1	260
breast_ptv_approved	0.97	0.98	0.97	236
chestwall_ctv_approved	1	1	1	42
chestwall_ptv_approved	1	1	1	5
combined_lungs_approved	1	1	1	174
combined_structure	0.73	0.8	0.67	6
contralateral_approved	1	1	1	80
control_structure	1	1	1	530
exclude	0.99	0.99	1	1152
heart_approved	1	1	1	166
imc_ctv_approved	1	1	1	45

imc_ptv_approved	1	1	1	5
left_lung_approved	1	0.99	1	172
right_lung_approved	1	1	1	172
scf_ctv_approved	0.99	1	0.98	59
scf_ptv_approved	0.5	1	0.33	3
tumourbed_ctv_approved	0.99	0.99	0.99	107
tumourbed_ptv_approved	1	1	1	7
micro avg	0.99	0.99	0.99	3255
macro avg	0.91	0.94	0.89	3255
weighted avg	0.99	0.99	0.99	3255
